# Outcomes of antibiotic treatment for respiratory infections in children an observational study in primary care

**DOI:** 10.1080/02813432.2024.2305929

**Published:** 2024-01-24

**Authors:** Linn Karin Tjalvin Alvsåker, Maria Fehn Stensen, Anders Batman Mjelle, Steinar Hunskaar, Ingrid Keilegavlen Rebnord

**Affiliations:** a Department of Medicine, University of Bergen, Bergen, Norway; b Department of Anaesthesiology and Intensive Care, Stavanger University Hospital, Stavanger, Norway; c National Centre for Emergency Primary Health Care, NORCE Norwegian Research Centre, Bergen, Norway; d Department of Global Public Health and Primary Care, University of Bergen, Bergen, Norway

**Keywords:** Adverse events, antibiotic prescribing, child, primary care, respiratory tract infections

## Abstract

**Background:**

Antibiotic resistance is an increasing global threat, accelerated by both misuse and overuse of antibiotics. Most antibiotics to humans are prescribed in primary care, commonly for respiratory symptoms, and there is a need for research on the usage of and outcomes after antibiotic treatment to counteract antibiotic resistance.

**Objective:**

To evaluate symptom duration, treatment length, and adverse events of antibiotic treatment in children.

**Design and Setting:**

Observational study at four out-of-hours services and one paediatric emergency clinic in Norwegian emergency primary care.

**Subjects:**

266 children aged 0 to 6 years with fever or respiratory symptoms.

**Main Outcome Measures:**

Duration of symptoms and absenteeism from kindergarten/school, treatment length, and reported adverse events.

**Results:**

There were no differences in duration of symptoms, fever or absenteeism when comparing the groups prescribed (30.8%) and not prescribed (69.2%) antibiotics. This lack of difference remained when analysing the subgroup with otitis media.

In the group prescribed antibiotics, 84.5% of parents reported giving antibiotics for 5-7 days, and 50.7% reported no difficulties. Adverse events of antibiotics were reported in 42.3% of the cases, the vast majority being gastrointestinal disturbances.

**Conclusion:**

Children with fever or respiratory symptoms experience similar duration of symptoms and absenteeism regardless of antibiotic treatment. A substantial number of parents reported adverse events when the child received antibiotics. Several parents experienced additional difficulties with the treatment, some ending treatment within day 4.

**Trial Registration Number:**

NCT02496559; Results.

## Key Points

Children with fever or respiratory symptoms treated at OOH services experience similar duration of symptoms or absenteeism, regardless of antibiotic treatment.

Parents often choose to end antibiotic treatment prematurely due to adverse events, bad taste, or that they find treatment unnecessary.

Children often experience adverse events when prescribed antibiotics, mainly gastrointestinal symptoms.

## Introduction

The discovery of antibiotics offered a possible treatment against former deadly infectious diseases and has prolonged the average human life expectancy [1]. Unfortunately, as medical practice changed to include antibiotics, the microbial genome also changed, and antibiotic resistance became an increasing global threat. In 2015, the World Health Organisation (WHO) formed the “Global action plan on antimicrobial resistance” [2]. To reach these goals, there is a need for research on all aspects of the usage of antibiotics, including children. In Norway, there has been published national guidelines with recommendations regarding indications for antibiotics, choice of antibiotics, and duration of treatment [3–4].

In Norway, 85% of antibiotics are prescribed in primary care, the most frequent users being young women, small children, and the elderly [5]. A substantial part of patients treated at out-of-hours (OOH) services are children with fever or respiratory tract symptoms. Young children are more prone to be infected by various infectious agents due to their underdeveloped immune system [6, 7]. Research suggests that more than half of such infections are viral [8–10]. Following the development of vaccines targeting bacteria such as S. pneumoniae and H. influenzae type B, bacterial infections have decreased [11–13]. Some bacterial infections may be more self-limiting than previously assumed. For instance, acute otitis media has shown only a slight, if any, shortening in the course of illness when treated with antibiotics [14, 15]. Nevertheless, the clinician must also consider that severe infections in children are more often caused by bacteria [8].

Antibiotics prescription is high across Europe, though with variations between countries. A recent study showed that a Norwegian child would, on average, be given 0.3 packages of antibiotics a year, compared to approximately 0.8 and 1.3 in Portugal and Hungary, respectively [7]. Antibiotics associated with treating respiratory tract infections accounted for as much as 70% of all antibiotics prescribed in Norway between 2005–2016 [9]. Findings suggest that antibiotic prescribing in the outpatient setting is influenced by many pressures, such as limited time for consultations, a wish for instant symptom relief, and expectations from the parents [10–12]. Inappropriate prescriptions may, to some extent, be explained by such factors.

Another aspect to consider when using antibiotics is the potential for adverse events. Most adverse events, such as vomiting, diarrhoea, rash, and pruritus, are not severe but can be uncomfortable enough to make patients stop the medication [13]. Research suggests that parents may be more concerned with their child experiencing adverse events than the potential of treatment failure [14]. Furthermore, parents often claim that their child is allergic, while a true penicillin allergy is found to be rare [15, 16]. Mislabelling of penicillin allergy increases the use of broad-spectrum antibiotics, leading to increased antibiotic resistance and greater occurrence of adverse events [16, 17].

Our study aimed to explore the course of fever or respiratory infections when children were treated with or without antibiotics at Norwegian OOH services, by observing duration of symptoms and absenteeism from kindergarten/school.

## Methods

This observational study included children from 0 to 6 years presenting with fever or respiratory symptoms at Norwegian OOH services. The data consists of clinical symptoms and signs collected by a nurse before the doctor’s consultation, the medical record, and questionnaires filled in by the parents before the consultation (additional file 1) and seven days after the consultation (additional file 2). One-third of the children were randomised to take a C-reactive protein (CRP) test before the consultation. The remaining two-thirds received a CRP test if the doctor ordered one on individual indication. The main results have been reported previously [18, 19]. The trial was registered on 14/07/2015, ClinicalTrials.gov, trial registration number NCT02496559. It was approved by the Regional Committee for Medical and Health Research Ethics (2012/1471/REK Vest).

### Inclusion and procedures

Participants were included at four different OOH services near Bergen and one paediatric emergency clinic at Haukeland University Hospital in Bergen during the winter seasons from January 2013 until May 2015. Children were included if they were aged 0 to 6 years, having fever and/or respiratory symptoms, and their parents consented to participate in the study.

The nurses selecting participants at the OOH services were informed about the study inclusion criteria and examination procedures. Two trained nurses were specially engaged at the paediatric emergency clinic for the project. The parents were approached by one of the nurses and invited to participate in the study. They gave their consent to fill in a questionnaire prior to the consultation, and a new questionnaire was sent by email after seven days. The nurses did a clinical examination of all children and a CRP test on every third child. After the consultation, the diagnosis and treatment were recorded from the medical record.

### Variables

Recorded variables from the medical history were, among others, the duration of the present illness and various symptoms. Variables from the nurse’s examination were vitals and general condition on a 3-point scale (normal, ill, and severely ill). From the medical records, we obtained information on diagnoses and prescriptions of antibiotics.

The authors designed a questionnaire using Qualtrics software (version 2014 of Qualtrics, copyright© 2014, Provo, UT). The primary outcome variables were duration of fever, cough, reduced general condition, dyspnoea, absenteeism from kindergarten/school, and occurrence of adverse events. If the child received antibiotics, the parents were asked about the duration of treatment and adverse events. These parents were asked to tick the appropriate box regarding their child’s treatment duration, choosing from a list of options: medication was taken without difficulty, or termination of treatment because of bad taste, allergic reaction/adverse event, healthy child, or the treatment was unnecessary. They could also state whether they had to get a new prescription. All these options could be ticked off for 0, 1, 2, 3, 5, or 7 days. Several parents ticked off multiple or all the reasons for ending treatment or changing antibiotics.

We chose to send out the questionnaire seven days after the inclusion, when most children were expected to have recovered and when parents still had the recovery and treatment fresh in mind. The guidelines at that time recommended a course of antibiotics for 7 days for otitis media, 7–10 days for pneumonia, and 10 days for tonsillitis [20].

### Statistical analysis

Background variables in the study population were analysed with cross-tabulation and means. Proportions were compared by Pearson χ2 tests, means by Independent Sample *t*-tests. An Independent Sample *t*-test was also used to compare mean symptom duration in days after the first consultation between patients prescribed or not prescribed antibiotics. Descriptive analyses were used to study the distribution of treatment duration and adverse events. The significance level was set at 5% (*p* < 0.05). The statistics were analysed using IBM SPSS Statistics 27.

## Results

### Baseline characteristics

**Figure 1.**
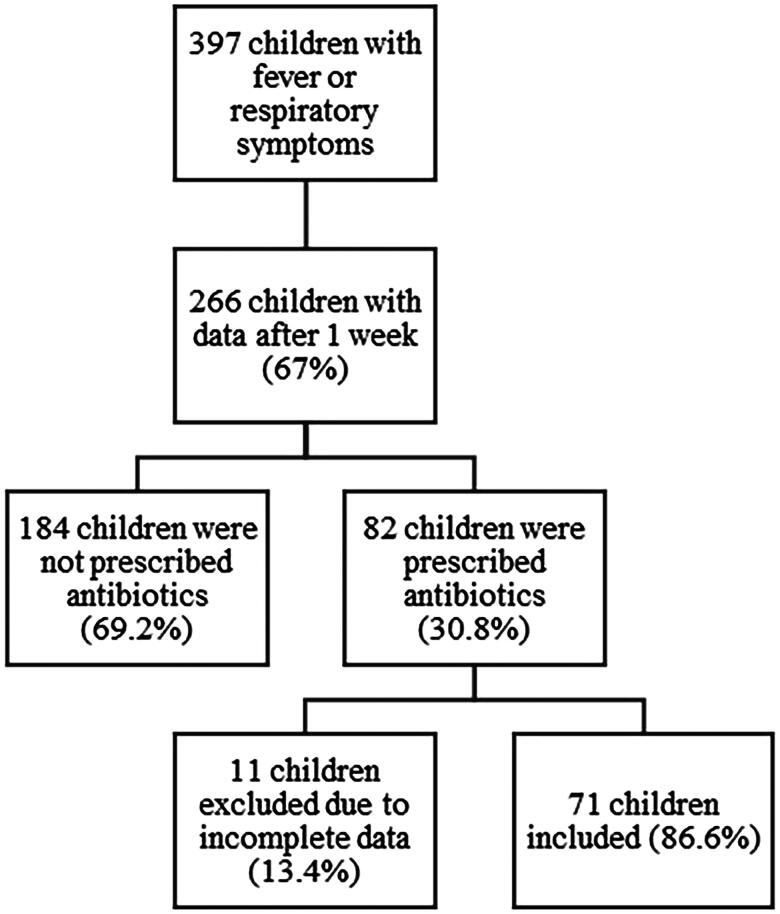
Flow chart over included patients in the study.

A total of 401 children were recruited for the study. Four patients left the clinic before the doctor’s consultation, leaving 397 children for analyses. The mean age at inclusion was 2.3 years, and 55.6% were males. The prescription rate of antibiotics at consultation was 23%, and the referral rate was 8%. Of the study population, parents of 266 (67%) children submitted a questionnaire seven days after the consultation and were included in our analysis as the responder group (Figure 1). This included 150 children (56.4%) from OOH services and 116 (43.6%) from the paediatric emergency clinic. Children in the responder group did not differ in age compared to the total study population, but there were slightly more male children (60%). The prescription rate was higher (30.8%) in the responder group, but the referral rate was identical (8%).

A comparison of baseline characteristics in the responder group is shown in Table 1 based on whether they were prescribed antibiotics or not at the consultation. Acute tonsillitis, otitis media, and pneumonia were significantly overrepresented in the group who received antibiotics. In the group who did not receive antibiotics, upper respiratory infections were significantly overrepresented. The majority of children were given penicillin V (52.4%). The remaining were given an antibiotic, either not specified (22.0%), amoxicillin (18.3%), a macrolide (4.9%) or clindamycin (2.4%).

**Table 1.** Distribution of the responder group (*n* = 266) and background variables.

	Total	Prescribed antibiotics	Not prescribed antibiotics	*p*-value
Participants, *n* (%)	266 (100)	82 (30.8)	184 (69.2)	
Age				
Mean (IQR)	2.4 (1.0 − 3.2)	2.6 (1.2 − 4.0)	2.3 (0.9 − 3.0)	0.119
Gender				
Boy, *n* (%)	160 (60.0)	55 (67.1)	105 (57.1)	0.124
Known chronic disease				
Asthma, *n* (%)	52 (19.5)	25 (30.5)	27 (14.7)	0.003
Symptoms^a^				
Fever, *n* (%)	230 (86.5)	76 (92.7)	154 (83.7)	0.048
Dyspnoea, *n* (%)	154 (57.9)	50 (61.0)	104 (56.5)	0.497
Earache^b^, *n* (%)	66 (25.0)	33 (40.2)	33 (18.1)	<0.001
Sore throat^b^, *n* (%)	147 (55.7)	52 (64.2)	95 (51.9)	0.064
General condition^c^				
Normal, *n* (%)	59 (22.3)	11 (13.4)	48 (26.2)	
Ill, *n* (%)	197 (74.3)	67 (81.7)	130 (71.0)	
Severely ill, *n* (%)	9 (3.4)	4 (4.9)	5 (2.7)	0.055
Hospital admissions				
Hospitalised, *n* (%)	21 (7.9)	7 (8.5)	14 (7.6)	0.796
Diagnoses				
Acute tonsillitis, *n* (%)	29 (10.9)	21 (25.6)	8 (4.3)	<0.001
Otitis media, *n* (%)	38 (14.3)	28 (34.1)	10 (5.4)	<0.001
Pneumonia, *n* (%)	12 (4.5)	10 (12.2)	2 (1.1)	<0.001
Upper respiratory infection, *n* (%)	152 (57.1)	19 (23.2)	133 (72.3)	<0.001
Asthma/bronchiolitis, *n* (%)	24 (9.0)	4 (4.9)	20 (10.9)	0.115
Other, *n* (%)	11 (4.1)	0 (0.0)	11 (6.0)	0.024

^a*^ *Symptoms last 24 h before the first consultation.*

^b^ *Total in responder group is 264.*

^c^*Total in responder group is 265*.

### Symptom duration

**Figure 2.**
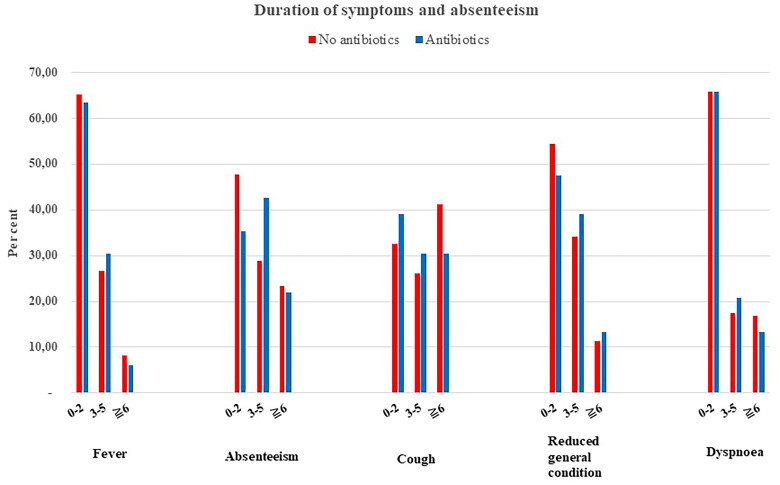
Proportion of patients (%) with symptoms and absenteeism from kindergarten/school for a given number of days, comparing the two groups.

No difference was found regarding symptom duration between the groups with and without antibiotic prescription, and the courses of illness regarding symptoms and absenteeism were quite similar (Figure 2). When performing sub-analyses of those diagnosed with tonsillitis, otitis media or pneumonia, which all often has a probable bacterial aetiology, there was still no difference (Table 2). Further, when analysing those with acute otitis media, which is a more defined diagnosis, we found the same results.

**Table 2.** Duration of symptoms and absenteeism in the two groups, shown for the total study population (*n* = 266), patients with probable bacterial infection (*n* = 79) and patients diagnosed with otitis media (*n* = 38).

	No antibiotics, mean (SD)	Antibiotics, mean (SD)	Difference (95% CI)	*p*-value
All patients (*n* = 266)	*n* = 184	*n* = 82		
Days with fever	3.17 (2.02)	3.27 (1.82)	−0.09 (−0.61–0.42)	0.72
Days with absenteeism^a^	4.03 (2.61)	4.49 (2.33)	−0.46 (−1.12–0.21)	0.18
Days with annoying cough	5.33 (2.91)	4.63 (3.06)	0.69 (−0.07–1.47)	0.08
Days with dyspnoea	3.25 (2.67)	2.99 (2.60)	0.26 (−0.43–0.96)	0.46
Days with reduced general condition	3.64 (2.19)	4.09 (2.19)	−0.45 (−1.02–0.12)	0.12
Patients with probable bacterial infection^b^	*n* = 20	*n* = 59		
Days with fever	3.20 (1.67)	2.97 (1.66)	0.23 (−0.62–1.09)	0.58
Days with absenteeism^a^	4.45 (2.74)	4.50 (2.28)	−0.14 (−1.38–1.09)	0.82
Days with annoying cough	4.85 (3.12)	4.46 (2.97)	0.39 (−1.16–1.95)	0.62
Days with dyspnoea	2.85 (2.80)	2.73 (2.43)	0.12 (−1.18–1.42)	0.85
Days with reduced general condition	3.85 (1.95)	3.92 (2.20)	−0.07 (−1.17–1.04)	0.91
Patients with otitis media (*n* = 38)	*n* = 10	*n* = 28		
Days with fever	3.00 (1.41)	2.89 (1.83)	0.11 (−1.19–1.41)	0.87
Days with absenteeism^a^	4.30 (2.36)	4.25 (2.15)	0.05 (−1.60–1.70)	0.95
Days with annoying cough	4.30 (3.34)	4.29 (3.13)	0.01 (−2.38–2.39)	0.99
Days with dyspnoea	2.30 (2.31)	2.64 (2.30)	−0.34 (−2.06–1.38)	0.69
Days with reduced general condition	3.70 (1.49)	3.93 (2.12)	−0.23 (−1.71–1.26)	0.76

^a^ *From kindergarten or school.*

^b^*Tonsillitis, otitis media and pneumonia*.

### Duration of antibiotic treatment

11 out of 82 patients prescribed antibiotics were excluded as their parents did not provide sufficient data on the duration of treatment and adverse events, leaving 71 patients for further analyses. Of these, five parents reported ending treatment on day 0, and six reported ending the treatment days 1-4. Duration of treatment is given in Table 3. A total of 60 parents (84.5%) reported giving antibiotics for 5-7 days, 36 (50.7%) without difficulty.

**Table 3.** Duration of treatment with antibiotics (*n* = 71).

	0 days, *n* (%)	1–4 days, *n* (%)	5–7 days, *n* (%)
Taken without difficulty	2 (2.8)	4 (5.6)	36 (50.7)
Terminated treatment^a^	3 (4.2)	2 (2.8)	9 (12.7)
Terminated treatment and prescribed new antibiotics^a^	0 (0.0)	0 (0.0)	15 (21.1)

^a^Due to bad taste, allergic reaction/adverse event, unnecessary treatment, or healthy child.

### Adverse events of antibiotic treatment

A substantial number of parents reported their child experiencing adverse events (42.3%), almost exclusively gastrointestinal disturbances (Table 4).

**Table 4.** Parent-reported adverse events when the child was prescribed antibiotics.

Adverse events reported, *n* (%)	
None	41 (57.7)
Gastrointestinal disturbances^a^	27 (38.0)
Rash	2 (2.8)
Other (sleepier than usual)	1 (1.4)
Total	71 (100)

^a^ *This includes diarrhoea, abdominal pain, or nausea*.

## Discussion

### Summary

In this study of children visiting OOH services with fever or respiratory symptoms and who were or were not prescribed antibiotics, no differences were found in symptom duration after the consultation. Most children were treated for 5-7 days without complications, while a few terminated their treatment early. A notable number of patients experienced adverse events when treated with antibiotics.

### Strength and limitations

A substantial number of parents answered the questionnaire after one week, giving us data on 266 children (67%). This was a higher response rate than we expected, based on experiences from surveys utilising data collected from individuals [21]. However, the study has some limitations. Firstly, our data was collected from 2013 to 2015, and there has been a reduction in antibiotic consumption in Norway since then [5]. However, the Norwegian Directorate of Health reports that a further reduction is needed [22]. The data is therefore still relevant, providing additional knowledge on the subject. Secondly, 13.4% provided incomplete questionnaires. Thirdly, we experienced some weaknesses with the questionnaire itself. No validated questionnaire was found. We therefore chose to use closed-ended items with tick-off boxes with yes/no or exact values to compare the answers easily. A few of the answers were contradictory concerning duration and reasons for treatment termination. Most parents reported several reasons for termination, which may be realistic as medicating children is often challenging. Furthermore, the information given in the second part of the study was only validated by the parents. As the parents knew whether their child was given antibiotics, this may have influenced how they interpreted the symptoms.

One of the study’s goals was to compare the outcomes of respiratory infections when children were prescribed or not prescribed antibiotics. We chose to focus on symptoms and absenteeism from kindergarten/school. Fever and other symptoms can often give rise to a lot of the subjective discomfort of a respiratory infection. Absenteeism is a more objective finding that often correlates to the degree of illness. The analyses relied on the assumption that the children in the two groups were identical, apart from taking antibiotics. However, the two groups were quite heterogeneous, encompassing different diagnosis spectrums and different degrees of severity. A plausible assumption would be that in the group who received antibiotics, there would have been patients with viral infections, patients with self-limiting bacterial infections, and bacterial infections requiring antibiotics. As our results showed no considerable improvements in symptoms or absenteeism, this may be an indication that some of the infections were viral and therefore not in need of antibiotic treatment anyway. That the two groups were not identical brings into question whether their course of illness can be rightly compared. However, analyses of background variables showed minimal differences beyond the fact that known asthma, fever, and earache were predictors for receiving antibiotics. Despite this limitation, the findings nevertheless indicate that the difference in time before recovery is minimal in the two groups. This is also reflected when analysing a more homogenous subgroup, such as those diagnosed with otitis media. Furthermore, the study did not explore patient compliance or whether the antibiotics prescribed were according to national guidelines, which also may have impacted the results.

### Comparison to existing literature

#### Symptom duration

Our results indicated no significant difference in the duration of absenteeism or symptoms such as fever in the two groups, consistent with previous findings [23–25]. Placebo-controlled trials have previously found antibiotics to have no significant effects on symptom duration [26, 27]. The lack of effectiveness of antibiotics found in both our data and others may suggest an overprescribing in paediatric populations and thus a potential for further reduction in prescribing.

One significant predictor for antibiotic prescription was earache. However, children diagnosed with otitis media did not have a reduction in symptom duration when receiving antibiotics. The diagnosis otitis media is often based on objective findings such as middle ear infusion, evidence of middle ear inflammation and bulging of the tympanic membrane [28]. The research on antibiotics used in this diagnosis group is nonetheless conflicting. Tapiainen et al. concluded that antibiotics reduced middle ear effusion and possible hearing impairment [30], whereas other studies have shown minimal or no significant effect on symptoms when compared with patients not given antibiotics [24, 29]. Our results provide support for no significant effect on symptom relief when using antibiotics for ear infections. Our material is too small, however, to analyse whether there are subgroups based e.g. on age, who could have benefitted more from antibiotics.

Diagnoses in primary care are often subjective, often symptom-based, such as cough or fever, or general, such as upper respiratory tract infection. Some of the diagnoses probably reflect that the doctor wants it to support the treatment given [31]. This makes it difficult to compare diagnoses directly and thus objectively assess whether antibiotics given are appropriate.

#### Duration of antibiotic treatment

Our results illustrate that most children receive antibiotics for 5–7 days without difficulty. Nonetheless, a substantial part of the participants experienced complications such as adverse events, allergic reactions, or bad taste. Szymczak et al. have previously shown that parents may be prone to end treatment early because they fear what they interpret as an allergic reaction [14]. It has been shown that many such parent-reported allergic reactions may instead be a symptom of a viral infection or an interaction between the medication and the virus [15, 17]. Adverse events and bad taste resulted in several parents requesting prescription for another type of antibiotics. It has been previously shown that these new prescriptions are more often broad-spectrum antibiotics, which in turn may lead to increased antibiotic resistance [32]. Our results support that too many children are prescribed antibiotics; some parents even stated ending treatment early as their child was healthy or that the treatment was unnecessary. Furthermore, the results indicate that the duration of treatment could be shortened. A recent study from Spain found that antibiotic treatment in adults may be equally effective when shortened, thus contributing less to antibiotic resistance [33]. These results may also apply to children.

#### Adverse events

Antibiotics are known to give rise to adverse events [25, 29, 30, 34–36]. Our study also indicated this, as 42.3% reported what they interpreted as an adverse event/allergic reaction in their child. As many as 38% stated that the child experienced gastrointestinal symptoms such as diarrhoea, abdominal pain, or nausea. Such complaints are strongly associated with antibiotic medication [25, 30, 34]. On the other hand, a recent study found no significant difference in reported adverse events when the child received antibiotics or not [37], implying that some of the adverse events reported by parents result from the infection itself.

### Implication for research and practice

Antibiotic use has been on the world’s agenda for a long time, and more knowledge is needed on when antibiotic treatment is appropriate within different patient groups to counteract the increasing trend in antibiotic resistance. This study provides additional data on the use and outcome of antibiotic treatment in children with fever or respiratory infections. From what we have experienced, there are few studies on this subject from primary care, proving a need for more research.

Our results indicate that the effect of antibiotics on symptom relief or duration of respiratory infection of unknown aetiology is highly questionable. This supports the fact that there is potential for better diagnostics and a higher threshold for treatment with antibiotics in children with upper airway infections. Measures should be taken to reduce the use of antibiotics, such as providing good information, making recontact an option, writing delayed prescriptions, and shortening treatment lengths. This has, to some extent, been implemented in Norwegian guidelines as the duration of antibiotic treatment has been shortened from 7 to 5 days for otitis media, 7 to 10 to 7 days for pneumonia, and 10 to 5 days for tonsillitis [3, 4]. As most antibiotics are prescribed in primary care, this is a beneficial place to focus our efforts.

## Conclusion

A considerable amount of antibiotics is prescribed for children with fever or respiratory symptoms in primary care. Our data showed similar duration of symptoms and absenteeism when comparing the children prescribed antibiotics with those not. This might indicate that some infections treated with antibiotics may be viral or self-limiting bacterial infections with no or limited effect of antibiotics. Many parents terminated treatment prematurely, attributing this to adverse events/allergic reactions, bad taste, or unnecessary treatment. The results support the ongoing efforts to reduce antibiotic use and the need for more knowledge on inappropriate prescriptions.

## Data Availability

The data underlying this article will be shared on reasonable request to the corresponding author.

## Additional files

- Questionnaire 1 (in Norwegian): Spørjeskjema til foreldre på legevakt.
- Questionnaire 2 (in Norwegian): *Spørjeskjema etter 7 dager.*
